# Exploring Physical Activity in Individuals With Type 2 Diabetes Mellitus and Lower Limb Complications: A Scoping Review

**DOI:** 10.1002/edm2.70084

**Published:** 2025-07-19

**Authors:** Bingyan Pang, Hannah Porter, Joanne A. McVeigh

**Affiliations:** ^1^ Curtin University Bentley Western Australia Australia; ^2^ Movement Physiology Laboratory University of Witwatersrand Johannesburg South Africa

**Keywords:** amputation, diabetic peripheral neuropathy, exercise, foot ulcer, physical activity, type 2 diabetes mellitus

## Abstract

**Aim:**

To synthesise contemporary evidence on physical activity (PA) levels in people with type two diabetes and lower limb complications (i.e., foot ulcer, peripheral neuropathy [PN], peripheral arterial disease and amputations).

**Methods:**

A scoping review following the JBI methodology was conducted using six databases: Medline, Embase, PubMed, Cochrane Library, SPORTDiscus and CINAHL. We included observational studies that primarily examined PA (all levels and types) in people with diabetes‐related lower limb complications. Studies published before December 2024 were included. We excluded reviews, intervention studies, and studies that examined the association between PA and T2DM risks. Findings were collated into tables and figures and reported narratively.

**Results:**

Sixteen studies met the inclusion criteria. Participants were reported to have PN, foot ulcer, peripheral arterial disease, or lower limb amputation. PA was assessed either by questionnaires or activity trackers. PA levels were reported as step count, duration of PA of different intensities, time spent in various postures, gait velocity, step rate and activity score. Mean daily step counts ranged between 1721 (amputation) and 7754 (PN). Mean moderate‐intensity PA was reported to be 2 min per day (amputation) to 37 min per day (PN).

**Conclusion:**

People living with diabetes‐related lower limb complications engage in low levels of PA. The findings suggest that people with more severe lower limb complications engage in less PA than those with less severe lower limb complications. Future research should standardise PA measurement in individuals with T2DM‐related lower limb complications and use the findings of this review to inform tailored, evidence‐based recommendations.

## Graphical abstract

People with diabetes‐related lower limb complications engage in low levels of physical activity. A linear reduction in daily step counts was observed in people with increasing severity of diabetes‐related lower limb complications. Standardised measurement of physical activity levels and investigation of the trajectory of physical activity are needed to develop evidence‐based recommendations in individuals living with diabetes‐related lower limb complications.

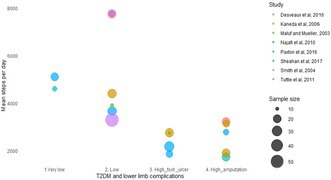

## 1 Introduction

Globally, over 462 million people are living with type 2 diabetes mellitus (T2DM), which is one of the leading causes of mortality and morbidity [1, 2]. In Australia, approximately one in 20 people are living with T2DM, with an associated annual healthcare cost of 1.9 billion dollars [3]. Individuals living with T2DM are at greater risk of developing lower limb complications such as peripheral neuropathy (loss of protective sensations) and diabetes‐related foot diseases compared to individuals without T2DM, leading to increased hospital admissions and lower limb amputations [2, 4, 5]. It is estimated that individuals with T2DM have a 19%–34% lifetime risk of developing foot wounds, and 65% of the healed diabetes foot wounds re‐occur within 5 years [6], contributing to the overall burden of diabetes [5]. In Australia, approximately 125 individuals are diagnosed with T2DM daily [3]. Strategies are needed to manage and prevent diabetes‐related lower limb complications to reduce the burdens of T2DM in individuals.

Physical activity (PA; any movement produced by the skeletal muscles [7]) is a known contributing factor to managing T2DM and preventing related health complications [5]. Regular and sufficient PA and reducing sedentary time are beneficial for glycaemic management, reducing the risk of cardiovascular mortality and overall health in individuals living with T2DM [8]. Diabetes‐related health complications and T2DM can be partially managed with PA through its effects on preventing and controlling insulin resistance [9]. Therefore, PA participation is often a key recommendation to enhance the health and well‐being of individuals living with T2DM [5, 7, 8]. The World Health Organisation recommends adults aged 18–64 years accumulate 150–300 min of moderate‐intensity PA or 75–150 min of vigorous‐intensity PA per week [7]. In addition, the World Health Organisation recommends people living with T2DM participate in a wide range of PAs, including aerobic activity, muscle strengthening, resistance training and interval training [8].

Although the literature supports PA participation in individuals with T2DM [2, 8], there are some inconsistencies in evidence for individuals with diabetes‐related lower limb complications, including foot ulcers, peripheral neuropathy (PN) and amputations. Participation in PA, especially weight‐bearing activities, may not be appropriate for this population [10, 11]. For instance, recent studies have reported that a reduction in weight bearing is generally prescribed for individuals living with active diabetes‐related foot diseases (i.e., active foot ulceration and/or Charcot foot) to alleviate pressure on the plantar foot and aid with healing [5, 11]. While some literature suggests that many individuals with lower limb amputations engage in low levels of PA [12] and are generally less active compared to those without such amputations [13], other studies have reported that some individuals with lower limb amputations maintain similar activity levels prior to amputation [14]. The existing literature on PA participation among individuals with diabetes‐related lower limb complications is limited and presents inconsistent findings [15].

Exploring PA levels in individuals with T2DM‐related lower limb complications is critical to developing tailored evidence‐based PA recommendations to help manage and prevent these complications [16, 17]. Existing literature tends to focus on the impacts of weight‐bearing activities in individuals living with T2DM‐related lower limb complications due to the nature of clinical recommendations to reduce such activities to aid plantar foot wound healing. Therefore, this scoping review aims to explore and synthesise literature on PA levels—considering all PA types—in people living with T2DM‐related lower limb complications.

## 2 Methods

This scoping review was guided by the JBI methodology for scoping review [18] and reported following the Preferred Reporting Items for Systematic Reviews extension for scoping reviews (PRISMA‐ScR) [19]. This scoping review is registered with the Open Science Framework: https://doi.org/10.17605/OSF.IO/RBGDE.

### 2.1 Search Strategy

A literature search was conducted in six databases, including Medline (Ovid), Embase (Ovid), PubMed, Cochrane, CINAHL and SPORTDiscus, in December 2024. Search terms were categorised into three concepts (See Table 1). The keywords and/or combinations of medical subject headings were modified in each database to optimise search strategies. No language or date restrictions were applied due to limited available evidence. Additionally, potentially relevant studies were identified by forward and backward searches and searching for the references of the included studies. A university librarian was consulted on the search strategies and selection of databases before the commencement of the search.

**TABLE 1:** Scoping review concepts and search terms.^a^

Concept 1	Concept 2	Concept 3
“type 2 diabetes” or “t2 diabetes” or t2dm or t2d or niddm or “non‐insulin dependent diabetes”	(foot or feet or toe or toes) ADJ3 (amputat* or ulcer* or disorder* or complaint* or deformit* or disabilit* or condition* or disease* or infect* or gangrene) OR “diabetic foot” or “diabetic feet” or “diabetes foot” or “charcot foot” or “charcot arthropathy” or “charcot neuroarthropathy” OR (foot or feet or toe or toes) ADJ8 (“peripheral neuropathy” or “peripheral nerve disease” or “diabetic neuropathy” or “peripheral arter** disease”)	“physical activit*” or exercise* or walk* or swim* or step*

^a^ Terms were connected with ‘OR’ and the concepts were combined with ‘AND’.

** ab.ti. were included at the end of the search phrase.

### 2.2 Selection Criteria

We included observational/cross‐sectional studies that reported on the population of interest (adults [18+ years of age] with T2DM who had a lower limb complication [e.g., foot ulcer, amputation, peripheral neuropathy]) and an outcome of interest (physical activity). Studies published before December 2024 were included. We considered all physical activity types. We excluded studies that were systematic reviews and intervention studies. Intervention studies inherently recruit participants with specific selection criteria, including (but not limited to) those below certain levels of physical activity, who do not currently engage in exercises, are above a certain weight, and have certain health conditions that could affect participants' physical activity levels [20]. In this review, we aimed to explore physical activity levels closer to free‐living conditions, without the bias of intervention‐related physical activity behaviour change. We excluded studies examining the association between PA and diabetes risk.

### 2.3 Study Selection

All database search findings were uploaded to Covidence software (https://www.covidence.org). Covidence automatically removed duplicates of studies. Two reviewers independently screened the titles and abstracts. Two reviewers independently screened the full texts. Any conflicts were resolved through discussion by referencing the predetermined research question, population and review outcomes.

### 2.4 Data Extraction and Analysis

One reviewer extracted data from the eligible studies using a dedicated data extraction table developed for this review within Covidence, and another reviewer checked the extracted data. The data on the relevant outcome variables were author(s), year of publication, sample size, country of publication, aims and objectives of the study, sample characteristics, measurement tool of PA, PA duration and intensity and key findings. The findings were collated into tables, summarised using figures and reported narratively.

In this review, the diabetes‐related foot complications were classified following the 2023 International Working Group Diabetic Foot Guidelines [21]. Four lower limb complication categories (according to severity) among individuals with diabetes were (1) very low: absence of peripheral arterial disease and peripheral neuropathy, (2) low: either peripheral arterial disease or peripheral neuropathy, (3) moderate: peripheral arterial disease and peripheral neuropathy, or peripheral neuropathy and foot deformities, or peripheral arterial disease and foot deformities and (4) high: peripheral neuropathy or peripheral arterial disease, plus one or more of the history of foot ulcer/lower limb amputation (toe, foot, or transtibial/leg)/end‐stage renal disease.

The quality of the studies was assessed by the JBI Critical Appraisal Analytical Cross‐Sectional Studies tool [22]. Two reviewers conducted the quality assessment to reduce potential bias.

## 3 Results

**FIGURE 1:**
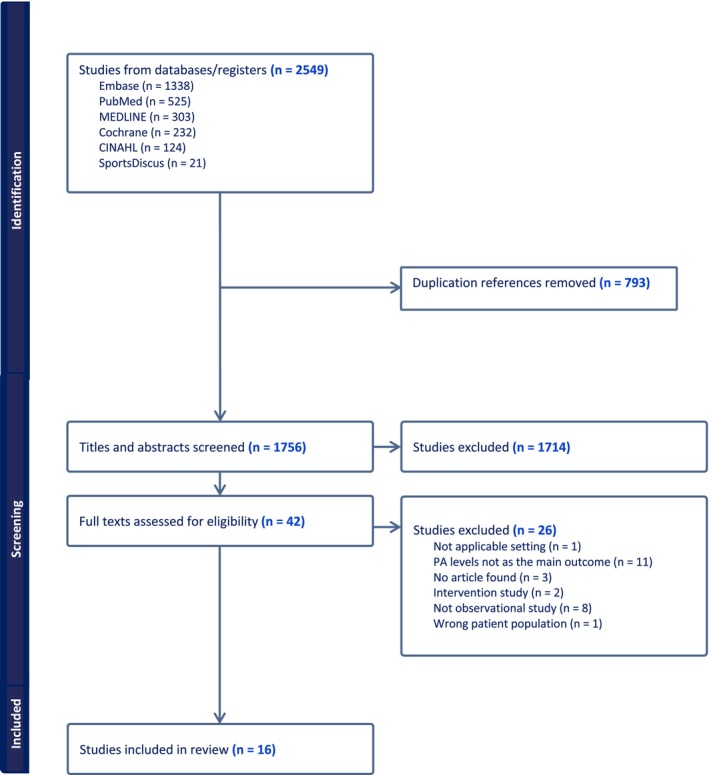
PRISMA chart.

The search from the six electronic databases obtained 2549 studies. A total of 793 duplicates were removed and 1756 titles and abstracts were screened. Forty‐two full texts were assessed and 16 studies [2, 4, 5, 23, 24, 25, 26, 27, 28, 29, 30, 31, 32, 33, 34, 35] were eligible to be included in this review. The publication dates of the included studies range from 2003 to 2024. The included studies were conducted in Australia (*n* = 4), Canada (*n* = 1), Nigeria (*n* = 1), UK (*n* = 4) and USA (*n* = 6). Figure 1 represents the PRISMA flowchart of the search results, and Table 2 shows the studies' characteristics. The quality assessment score for the included studies is shown in Table 2. For most studies, the study design, objective, method of participant selection and outcomes were clearly defined.

**TABLE 2:** An overview of the characteristics of the 16 studies included in this review.

Authors, year	Country	Study aim/s	Lower limb complications	Other co‐morbidities	Method of PA measurement	Location of device	Device wear times	PA outcomes assessed	Quality assessment
Adeniyi et al., 2015	Nigeria	To investigate the links among physical activity and selected indices of neuromusculoskeletal disorders among a group of patients with T2DM	PN, foot deformities	Hypertension, osteoarthritis, eye problems, back pain	Baecke's Habitual PA Questionnaire	—	—	Index in work activity, sports activity, leisure activity	6
Crews et al., 2017	USA	To assess the feasibility of objectively, synchronously, and continuously monitoring finely detailed PA and its location of occurrence in individuals at risk and with active diabetic foot ulcers	Diabetic foot ulcers	—	Accelerometer (PAMSys), GPS logger	PAMSys in a pouch of T‐shirt. GPS on a belt or pocket.	72 h except while bathing	Time in standing, walking, lying, sitting	6
Delpierre et al., 2023	UK	To investigate the short‐term impact of non‐removal offloading device on physical activity and diabetic foot ulcer related quality of life in a small sample of community‐dwelling people living with diabetic foot ulcer	Diabetic foot ulcers	—	Global Physical Activity Questionnaire	—	—	Physical activity categories: low, moderate, high according to the questionnaire manual	7
Desveaux et al., 2016	Canada	To determine whether adults with diabetes and transtibial amputations meet recommended guidelines for activity intensity and daily step counts	Transtibial amputations	Arthritis, cardiovascular conditions, dyslipidaemia, respiratory conditions	Accelerometer (step activity monitor)	Ankle of intact limb	9 consecutive days to ensure at least 5 days of accurate data collection	Steps per day and minutes of MVPA/week	7
Johnson et al., 2019	Australia	To describe self‐reported physical activity participation of community‐dwelling Australian adults with diabetes and complications	PN, diabetic foot ulcer	Cardiovascular disease, retinopathy/nephropathy	International Physical Activity Questionnaire	—	—	Walking, sitting, moderate/vigorous physical activity minutes per week	7
Kanade et al., 2006	UK	To study the capacity and performance in relation to walking activity and its impact on the plantar tissues across the various groups with PN	PN, diabetic foot ulcers, foot amputation, transtibial amputations	—	Accelerometer (step activity monitor), gait velocity	Above ankle	8 continuous days, excluding bathing or swimming	Gait velocity, average daily strides	7
Lee et al., 2019	Australia	Investigate moderate‐to‐vigorous‐intensity physical activity performed by people with diabetic foot ulcer and to compare it to those with PN without foot ulcer history and those with diabetes without PN or foot ulcer in their everyday free‐living environments	PN, diabetic foot ulcer	Cardiovascular disease, chronic kidney disease, dyslipidaemia	SenseWear Armband	Mid‐upper arm (no arm specified)	5 continuous days. Worn at all times except in water based activities	Minutes per bout of MVPA, intensity per bout of MVPA (METs) and accumulated minutes daily time spent doing MVPA	7
Maluf and Mueller, 2003	USA	To compare the amount of weight‐bearing activity and estimates of cumulative plantar tissue stress between participants with diabetes mellitus and peripheral neuropathy and a history of recurrent plantar ulcers	PN, diabetic foot ulcer	—	Accelerometer (step activity monitor)	Above ankle	7 consecutive days	Strides per day	7
Murphy et al., 2024	Australia	To evaluate an approach to collect and analyse device‐derived 24‐h use of time data in people with a diabetic foot ulcer	Diabetic foot ulcer	—	Accelerometer (GENEActiv)	Non‐dominant wrist	14 continuous days, removed during water submersion	Duration in sedentary time, sleep, light physical activity, MVPA	7
Najafi et al., 2010	USA	To describe the quality and quantity of activities of daily living in PN patients	PN	—	Accelerometer (PAMSys)	On t‐shirt middle near sternum	48 h, remove shirt prior to bathing	Time spent in walking, standing, sitting, lying	6
Neal et al., 2024	UK	To provide detailed understanding of the physical activity intensity and sleep profile, assessed with accelerometers, for individuals with active diabetic foot ulcers	Diabetic foot ulcer	Cardiovascular conditions	Accelerometer (GENEActiv)	Non‐dominant wrist	8 continuous days	Average acceleration, durations of physical activity	6
Paxton et al., 2016	USA	Characterise PA in people with diabetes with and without transtibial amputation	Transtibial amputation		Accelerometer (ActiGraph GT3X)	Waist at midaxillary line	10 consecutive days	Steps per day, light, moderate, vigorous PA	5
Perks et al., 2023	UK	To quantify differences in key metrics characterising physical activity and physical function in people with T2DM, with and without peripheral arterial disease	Peripheral arterial disease	Angina, angioplasty, heart failure, hypercholesterol‐aemia, myocardial infarction, stroke, thyroid disorder	Accelerometer (GENEActiv)	Non‐dominant wrist	8 continuous days	Average acceleration, time in light PA, MVPA in at least 1‐min bouts.	6
Sheahan et al., 2017	Australia	Investigate multiple daily activity outcomes in patients with diabetic foot ulcer compared to PN and diabetes controls in their free‐living environments	PN, foot deformity, peripheral arterial disease, diabetic foot ulcer	Cardiovascular diseases, chronic kidney diseases, dyslipidaemia, hypertension, previous amputation	SenseWear Armband	Triceps at mid‐point of humerus (no arm specified)	7 continuous days	Steps per day, average METs, PA	6
Smith et al., 2004	USA	To examine the relationship between self‐reported physical activity using short form 36 and actual step counts in men with diabetes	PN	Lower limb amputation	Accelerometer (step activity monitor)	Right ankle	14 continuous days, except sleeping or bathing	Steps per day, walking intensity, durations of PA	7
Tuttle et al., 2011	USA	To characterise PA levels (average daily step count) in a sample of people with diabetes and PN and to determine the relationship between step count and intermuscular adipose tissue volume, muscle performance and physical function	PN	—	Accelerometer (step activity monitor)	Ankle	9 consecutive days	Steps per day	7

Abbreviations: METs, metabolic equivalent tasks; MVPA, moderate to vigorous physical activity; PA, physical activity; PN, peripheral neuropathy; T2DM, type 2 diabetes mellitus.

Table 3 shows the characteristics of the participants in the included studies. The age range of the participants was around 55–65 years. The majority (*n* = 14, 87.5%) of the included studies had more men than women. Participants were reported to have T2DM‐related lower limb complications, including PN (*n* = 338), foot deformities (*n* = 212), foot ulcers (*n* = 225), peripheral arterial disease (*n* = 47), or lower limb amputation (*n* = 73). The sample size in each study ranged between 10 and 264 and ~80% of the studies had a sample size of less than 50.

**TABLE 3:** Participant demographics of the included studies.

Authors, year	Diabetes and lower limb complications	Number of participants (*n*)	Gender		Mean age ± SD in years
			Women (*n*)	Men (*n*)	
Adeyini et al., 2015	T2DM only	—	—	—	—
	T2DM + foot deformities (79%)	212	—	—	—
	T2DM + PN (26%)	69	—	—	—
	Total	264	177	87	59 ± 6.8
Crews et al., 2017	T2DM only	5	5	0	55 ± 11
	T2DM + foot ulcer	5	1	4	55 ± 5
Delpierre et al., 2023	DM^a^ + foot ulcer + amputation (44%)	18	9	9	58 ± 10.1
Desveaux et al., 2016	T2DM + transtibial amputation at baseline	22	6	16	63 ± 12
	T2DM + transtibial amputation at follow‐up	15	5	10	61 ± 12
Johnson et al., 2019	T2DM + PN	98	—	—	—
	T2DM + foot ulcer	31	—	—	—
	Total^b^	240	101	139	69 ± 10.5
Kaneda et al., 2006	T2DM + PN	23	3	20	64 ± 5.8
	T2DM + foot ulcer	23	4	19	60 ± 9.6
	T2DM + foot amputation	16	1	15	62 ± 8.8
	T2DM + transtibial amputation	22	2	20	63 ± 6.1
Lee et al., 2019	T2DM	15	8	7	59 ± 11
	T2DM + PN	23	9	14	68 ± 9
	T2DM + foot ulcer	27	6	21	56 ± 11
	Total	65	23	42	61 ± 11
Maluf and Mueller, 2003	T2DM + PN	10	3	7	58 ± 6.2
	T2DM + foot ulcer	10	3	7	55 ± 11.3
Murphy et al., 2024	T2DM + foot ulcer	25	3	23	68 ± 9.2
Najafi et al., 2010	T2DM + PN	13	—	—	59 ± 8
Neal et al., 2024	T2DM	561	200	361	64 ± 8.2
	T2DM + foot ulcer	34	4	30	59 ± 8.9
Paxton et al., 2016	T2DM	11	2	9	62 ± 8.3
	T2DM + transtibial amputation	22	3	19	61 ± 15.3
Perks et al., 2023^d^	T2DM	689	243	446	65.0
	T2DM + peripheral arterial disease	47	10	37	65.0
Sheahan et al., 2017	T2DM	20	9	11	60.0 ± 10.0
	T2DM + PN	23 (4 + foot deformities)	8	15	68.0 ± 9.0
	T2DM + foot ulcer	30 (22 + foot deformities)	6	24	57.0 ± 11.0
	T2DM + foot ulcer + no amputation^c^	17	4	12	55 ± 13
	T2DM + foot ulcer + minor amputation^c^	13	1	12	58 ± 10
Smith et al., 2004^d^	T2DM + PN	57	—	67	68
Tuttle et al., 2011	T2DM + PN	22	7	15	64.5 ± 12.7

Abbreviations: PN, peripheral neuropathy; T2DM, type 2 diabetes mellitus.

^a^ Study included a sample of participants with type 1 (39%) and type 2 diabetes (61%). There was no separate reporting of genders in the types of diabetes.

^b^ Study included participants with type 1 (4%) and type 2 diabetes (96%).

^c^ Sub‐group of the T2DM + foot ulcer participants.

^d^ The authors reported mean age and age range in this paper. The standard deviation of the participants' age was not reported.

The measurement tool for assessing PA levels differed across studies (see Table 2). PA levels were assessed either by questionnaires (*n* = 3) or activity trackers (*n* = 13). Of the 13 studies that used activity trackers to measure PA, the locations of activity tracker placement were ankle (*n* = 5), arm (*n* = 1), non‐dominant wrist (*n* = 3), shirt pocket (*n* = 2), upper arm (*n* = 1) and waist (*n* = 1). The activity tracking devices included an ActiGraph GT3X (*n* = 1), GeneActiv (*n* = 3), PAMSys (*n* = 2), SenseWear armband (*n* = 2) and step activity monitor (*n* = 5). Physical activity levels were reported as activity scores, energy expenditure, gait velocity, minutes PA of different intensities per day and/or week, steps per day, step rate, strides per day, and time spent in various postures.

**FIGURE 2:**
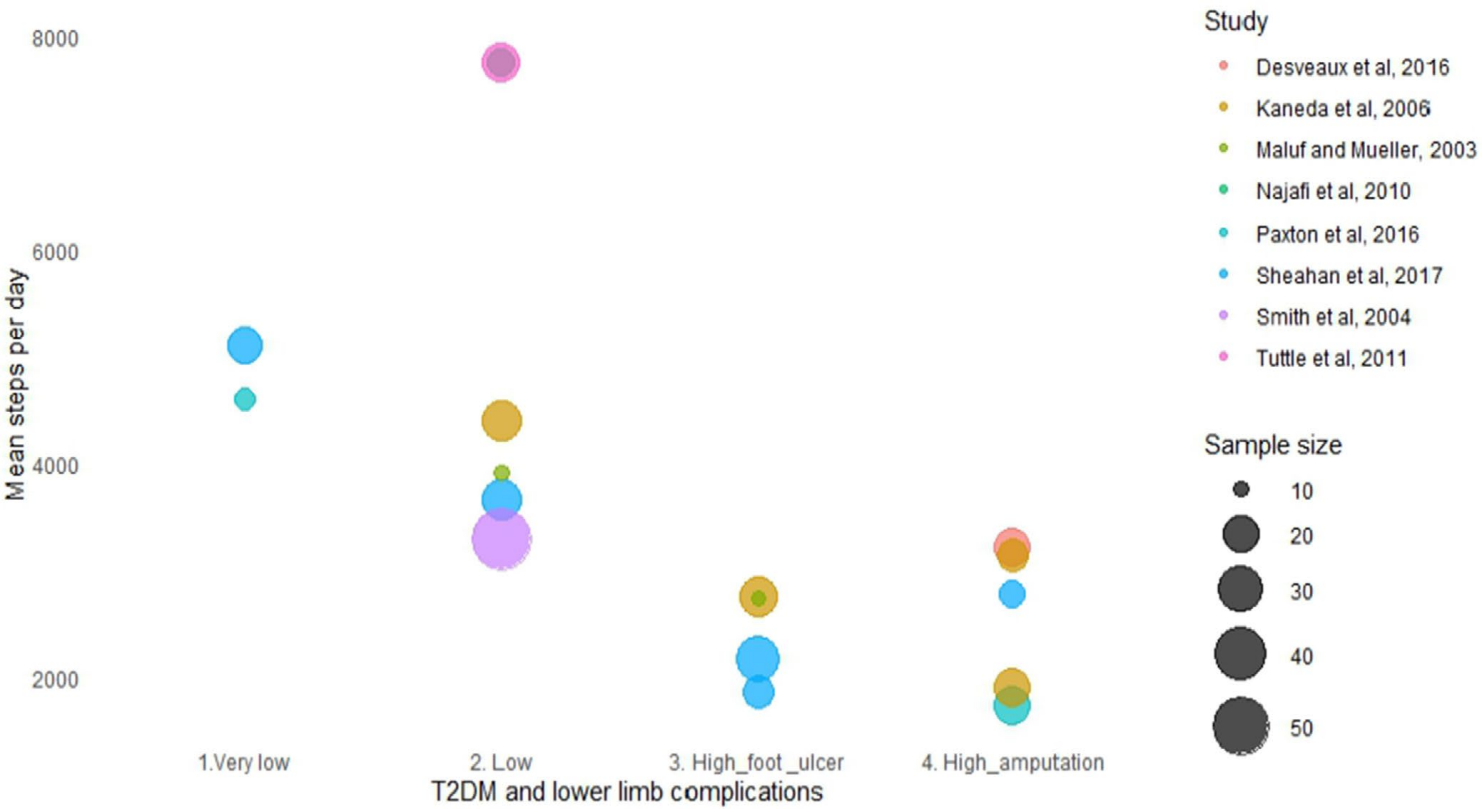
Daily steps reported in eight studies. The *x*‐axis presents the T2DM and lower limb complications based on the classification of the 2023 International Working Group Diabetic Foot Guidelines [21]: Very low = ones without the presence of peripheral arterial disease and no peripheral neuropathy; low = ones with either peripheral arterial disease or peripheral neuropathy; high_foot_ulcer = high, ones with peripheral neuropathy or peripheral arterial disease, plus one or more of the history of foot ulcer; high_amputation = high, ones with peripheral neuropathy or peripheral arterial disease, plus one or more of the history of lower limb amputation (toe, foot, or transtibial/leg).

Eight studies presented PA outcomes as steps per day, and the mean steps per day reported in these studies are presented in Figure 2. A general trend of fewer mean steps per day with more severe lower limb complications was observed. People with very low T2DM‐related lower limb complications were reported to engage between 4500 and 5500 steps per day. People with high T2DM‐related lower limb complications (amputations) were reported to engage in as low as 1721 mean steps per day.

Other PA outcomes from 14 studies (excluding steps per day) are presented in Table 4. Four studies presented PA outcomes in average activity duration (minutes) and intensities per day. Participants with low T2DM‐related lower limb complications were reported to engage in 37 min of moderate‐intensity PA/MVPA per day. Participants with high T2DM‐related lower limb complications were reported to engage in less than 10 min of moderate‐intensity PA/MVPA per day. Overall, less physical activity was observed in participants with higher lower limb complications.

**TABLE 4:** Main physical activity outcomes reported in 14 studies.

Authors, year	Diabetes and lower limb complications	Physical activity outcomes	Physical activity outcome units
Adeyini et al., 2015	T2DM ± PN or foot deformities^a^	6.1 ± 1.56 total activity score per week	Activity score per week
Crews et al., 2017	T2DM only	152 ± 95 min per day standing and walking at home 126 ± 90 min per day standing and walking outside the home 296 min per day away from home (combined walking, standing, sitting, and lying)	Time duration in minutes in activity postures per day
	T2DM + foot ulcer	157 ± 155 min per day standing and walking at home 55 ± 31 min per day standing and walking outside the home 110 min per day away from home (combined walking, standing, sitting, and lying)	
Delpierre et al., 2023	DM^b^ + foot ulcers + amputation	Low activity (67%), moderate activity (17%), high activity (17%)	The intensity of activity in the percentage of the day
Desveaux et al., 2016	T2DM + leg amputation at base line	31 ± 52 min MVPA per week	Time duration in minutes in MVPA per week
	T2DM + leg amputation at 6 months follow‐up	24 ± 41 min MVPA per week	
Johnson et al., 2019	DM^c^ ± PN ± foot ulcers	340 ± 180 min sitting per week 272 ± 393 min walking per week 307 ± 380 min moderate intensity physical activity per week 35 ± 98 min vigorous intensity physical activity per week	Time duration in minutes in activity postures per week. Time duration in minutes in physical activity intensities per week
Kanede et al., 2006	T2DM + PN	1.1 ± 0.2 gait velocity metres per second	Gait velocity meters per second
	T2DM + foot ulcer	0.9 ± 0.3 gait velocity metres per second	
	T2DM + foot amputation	0.9 ± 0.2 gait velocity metres per second	
	T2DM + leg amputation	0.7 ± 0.2 gait velocity metres per second	
Lee et al., 2019	T2DM	728 ± 123 min sedentary activity per day 40 (21–65) min MVPA per day	Time duration in minutes in physical activity intensities per day.
	T2DM + PN	720 ± 140 min sedentary activity per day 37 (19–76) min MVPA per day	
	T2DM + foot ulcer	796 ± 120 min sedentary activity per day 36 (17–64) min MVPA per day	
Murphy et al., 2024	T2DM + foot ulcer	485 min sleep per day 747 min sedentary behaviours per day 172 min light intensity physical activity per day 13 min MVPA per day	Time duration in minutes in physical activity intensities per day
Najafi et al., 2010	T2DM + PN	44.3% ± 8.1% of time lying per day 37.3% ± 6.3% of time sitting per day 13.5% ± 5.3% of time in standing per day 6.1% ± 3.1% of time walking per day	Time duration in minutes in activity postures per day.
Neal et al., 2024	T2DM	21.9 mg average acceleration in 24 h 400 (393–408) min sleep per day 755 (744–765) min sedentary behaviours per day 179 (173–185) min light intensity physical activity per day 18 (16–21) min MVPA per day	Time duration in minutes in physical activity intensities per day
	T2DM + foot ulcer	16.9 mg average acceleration in 24 h 396 (372–421) min sleep per day 848 (812–883) min sedentary behaviours per day 138 (119–157) min light intensity physical activity per day 9 (5–17) min MVPA per day	
Paxton et al., 2016	T2DM	82.1% ± 6.8% of time in sedentary behaviours per day 14.4% ± 7.0% of time in light intensity physical activity per day 2.7% ± 1.6% of time in moderate intensity physical activity per day 0% of time in vigorous intensity physical activity per day	Time in physical activity intensities in percentage of day
	T2DM + leg amputation	93.3% ± 5.8% of time in sedentary behaviours per day 13.2% ± 34.0% of time in light intensity physical activity per day 2.0% ± 5.0% of time in moderate intensity physical activity per day 0% of time in vigorous intensity physical activity per day	
Perks et al., 2023	T2DM	21.2 mg (17.3–25.8) average acceleration in 24 h 755 (692–809) min sedentary behaviours per day 171 (141–209) min light intensity physical activity per day 15 (7–30) min MVPA per day	Time duration in minutes in physical activity intensities per day
	T2DM + peripheral arterial disease	19.2 mg (12.9–22.9) average acceleration in 24 h 801 (715–861) min of sedentary behaviours per day 154 (110–208) min light intensity physical activity per day 7 (2–14) min MVPA per day	
Sheahan et al.,^e^ 2017	T2DM	47 (26–73) min moderate‐intensity physical activity per day	Time duration in minutes in physical activity intensities per day
	T2DM + PN	37 (18–60) min moderate‐intensity physical activity per day	
	T2DM + foot ulcer	40 (20–69) min moderate‐intensity physical activity per day	
	T2DM + foot ulcer + no amputation^d^	29 (16–42) min moderate‐intensity physical activity per day	
	T2DM + foot ulcer + minor amputation^d^	69 (47–152) min moderate‐intensity physical activity per day	
Smith et al., 2004	T2DM + PN	1094 ± 133 min with no steps per day 86 ± 44 min with very low step rate (1–2 steps/min) per day 140 ± 51 min with low step rate (3–10 steps/min) per day 103 ± 60 min with moderate step rate (11–30 steps/min) per day 17 ± 24 min with high step rates (31–60 steps/min) per day	Time duration in minutes in physical activity intensities per day

*Note:* Studies that measured step count are shown in Figure 2.

Abbreviations: MVPA, moderate to vigorous physical activity; PN, peripheral neuropathy; T2DM, type 2 diabetes mellitus.

^a^ Authors did not report physical activity scores separately for the different lower limb complication groups.

^b^ Study included a sample of participants with type 1 (39%) and type 2 diabetes (61%). There was no separate reporting of genders in the types of diabetes.

^c^ Study included participants with type 1 (4%) and type 2 diabetes (96%). There was no separate reporting of activities in participants with different lower limb complications.

^d^ Sub‐group of the T2DM + foot ulcer participants.

^e^ The moderate‐intensity physical activity minutes in this study are based on energy expenditure (> 3.0 METs).

## 4 Discussion

This scoping review synthesised literature on PA levels in people living with T2DM‐related lower limb complications, including PN, diabetic foot ulcer, peripheral arterial disease and lower limb amputations. The findings suggest that most individuals living with T2DM and lower limb complications are not meeting the recommended levels of PA [36]. A general trend of less PA was observed with more severe lower limb complications regardless of the method of PA measurement and reported PA outcomes. A linear reduction in step counts with people with increasing severity of lower limb complications was also observed in this review. People with T2DM‐related high lower limb complications (e.g., foot ulcers and/or amputations) are the least physically active group, engaging in < 10 min of MVPA per day.

Most people with T2DM‐related lower limb complications engage in < 5500 steps per day, less than the recommended 7000 steps per day [36]. The number of steps per day appeared to decrease in those with higher lower limb complications, suggesting a negative linear relationship between steps per day and T2DM‐related lower limb complications. However, all the included studies were cross‐sectional, and causal relationships could not be determined. It is possible that people with less severe T2DM‐related lower limb complications were more physically able to be more active compared to people with higher complications. Two studies [30, 35] reported participants with low T2DM‐related lower limb complications engaged in a mean of ~7500 steps per day, suggesting they could be meeting the recommended PA levels [36]. However, both studies reported much higher standard deviations (> 4000 steps per day) compared to the other included studies in this review, suggesting that those with extremely high step counts may have inflated the reported mean of ~7500 steps per day. In addition, one group of participants with > 8000 steps per day was also younger (mean age = 55 years) [35] compared to participants with fewer steps per day. People with diabetes‐related foot ulcers were reported to have significantly fewer steps per day compared to ones without foot ulcers, and similar steps per day to ones with amputations. This corresponds with clinical recommendations [37] for plantar foot ulcer healing, such that individuals should reduce their time spent weight‐bearing (taking steps) and offloading pressure to the plantar feet when possible. However, the reduction in steps per day and being less physically active could have negative impacts on people's T2DM management.

The time spent in different PA intensities appears to follow the same pattern as mean step counts per day, suggesting that people with very low and low T2DM‐related lower limb complications engage in longer time and higher intensity PA than those with more severe complications. However, it is challenging to harmonise and compare the reported findings in terms of time duration and intensity of PA due to the differences in measurement tools. Three studies [23, 25, 26] used questionnaires to assess PA levels, of which only one [4] of the three studies reported specifically on the duration and intensity of PA participation. In one study [23], PA was presented in domains of work, sports and leisure in a scale figure specific to the measuring questionnaire, making it challenging to extract the actual duration and intensity of PA. The remaining 13 studies used metrics derived from activity trackers placed on shirt pockets [5, 24], ankles [4, 27, 28, 34, 35], upper arms [2], arm [33], non‐dominant wrists [5, 29, 32] and waist [31]. Due to the differences in activity tracker placement and proprietary algorithms of some devices, the duration and intensity of PA reported in the studies had to be interpreted with caution in people with T2DM‐related lower limb complications.

People with low to high T2DM‐related lower limb complications reported predominantly spending their wake time in sedentary and some light‐intensity PA [2, 5, 24, 25, 26, 29, 31, 32]. This finding aligns with previous literature that reported people with peripheral arterial disease rarely exceeded light intensity in their activities [38]. Although light‐intensity PA is an important element of an active lifestyle, higher‐intensity PA could be more beneficial to elicit health impacts when the individual is physically able to do so [7]. Participants without T2DM‐related lower limb complications who engage in short bursts of high‐intensity PA throughout the day were reported to have positive impacts in managing T2DM and cardiovascular health [39, 40]. To ensure that the higher‐intensity movements do not impact plantar foot wound healing, research could investigate the types, feasibility, and safety of short bursts of high‐intensity PA in people with T2DM‐related lower limb complications to improve T2DM management and diabetes‐related health outcomes. Future research could also investigate barriers people with T2DM‐related lower limb complications experience when participating in PA to ensure the strategies to promote PA are appropriate and acceptable to this population.

## 5 Strengths and Limitations

This scoping review included observational studies that provided snapshots of PA levels of people with T2DM‐related lower limb complications. The focus on observational studies allowed authors to explore and synthesise evidence on PA levels in the population of interest, reflecting patterns in real‐world settings and avoiding potential artificial inflation of PA in intervention studies. The scoping review also included participants with different severities of T2DM‐related lower limb complications, providing insights into a reduction in PA levels with increasing severity of the complications. The findings of this review could form the basis for future studies in developing tailored recommendations to promote PA in people living with T2DM‐related lower limb complications.

Several limitations should be considered when interpreting the findings of this review. This review considered PA levels derived from measurement tools described in the included studies. The heterogeneity in the measurement tools used to assess PA across the studies presented inconsistent outcome metrics, making it challenging to harmonise the findings. In several studies, the proprietary algorithms of the activity tracking devices were used to derive PA levels. It was unclear whether the algorithms were validated for the study population of interest. The variety in measurement tools and derived metrics such as activity scores, energy expenditure, gait velocity, minutes PA of different intensities per day and/or week, steps per day, step rate, strides per day, and time spent in various postures presented challenges to drawing consistent conclusions across studies. In this review, the diabetes‐related foot complications classifications were based on the 2023 International Working Group Diabetic Foot Guidelines [21]. The classification does not distinguish between high‐risk people with foot ulcers and amputation. The findings of the review were presented separately for high‐risk foot ulcer and high‐risk amputation for the demonstration of reduction in physical activity and severity of the complications. Furthermore, the sample size of the participants with low to high T2DM‐related lower limb complications in the included studies rarely exceeded 50 participants. The small samples may not adequately represent the diversity of the population with T2DM, limiting the external validity and applicability of findings to broader clinical or research contexts. With increasing daily diagnoses of T2DM and potential lower limb complications, more research on PA levels in this population is needed to determine optimal levels of PA to manage T2DM and prevent lower limb complications.

## 6 Implications and Future Research Recommendations

The findings from this scoping review indicated the need for standardised measurement of 24‐h PA levels and investigation of the trajectory of PA to develop evidence‐based PA recommendations and tailored interventions in individuals living with T2DM‐related lower limb complications. Such recommendations and interventions need to consider the potential lower PA level baseline in this population compared to the general population, the safety of PA and options of PA for individuals with active foot ulcers that need to reduce plantar foot pressure for wound healing, consider the need to monitor glycaemic control before/during/after participation in PA, as well as returning to activity after an episode of lower limb complications. Tailored interventions should encourage participation in individualised and meaningful PA options, considering the severity of lower limb complications. Future research should consider co‐designing PA interventions with the population of interest to optimise feasibility and applicability and develop translatable PA monitoring metrics for people living with T2DM‐related lower limb complications.

## 7 Conclusion

This scoping review synthesised evidence of PA levels in individuals with T2DM‐related lower limb complications. The findings suggest this population engages in low levels of PA. The review shows that individuals with T2DM‐related lower limb complications do not meet the PA participation recommendations, and decreasing PA levels become evident as lower limb complications increase in severity. A range of physical activity measurement tools was identified. Physical activity outcomes were presented as activity scores, energy expenditure, gait velocity, minutes of PA in varying intensities, steps per day, step rate, strides rate, and time spent in various postures. This review highlighted the need for standardised measurement of PA and better insights into the 24‐h pattern of PA behaviours in this population. Future research is needed to develop evidence‐based and tailored recommendations for PA participation in this population to prevent lower limb complications and burdens of T2DM.

## Author Contributions

**Bingyan Pang:** conceptualization (lead), data curation (lead), formal analysis (lead), funding acquisition (lead), investigation (lead), methodology (lead), project administration (lead), supervision (lead), visualization (lead), writing – original draft (supporting), writing – review and editing (lead). **Hannah Porter:** conceptualization (supporting), data curation (supporting), formal analysis (supporting), investigation (equal), project administration (equal), visualization (supporting), writing – original draft (equal), writing – review and editing (supporting). **Joanne A. McVeigh:** conceptualization (supporting), formal analysis (supporting), funding acquisition (supporting), methodology (supporting), project administration (supporting), supervision (equal), visualization (supporting), writing – review and editing (equal).

## Conflicts of Interest

The authors declare no conflicts of interest.

## Data Availability

The authors have nothing to report.
